# Neural connectivity patterns explain why adolescents perceive the world as moving slow

**DOI:** 10.1038/s42003-024-06439-4

**Published:** 2024-06-22

**Authors:** Foroogh Ghorbani, Xianzhen Zhou, Nasibeh Talebi, Veit Roessner, Bernhard Hommel, Astrid Prochnow, Christian Beste

**Affiliations:** 1 Cognitive Neurophysiology, Department of Child and Adolescent Psychiatry, Faculty of Medicine, TU Dresden, Schubertstrasse 42, 01307 Dresden, Germany; 2 School of Psychology, Shandong Normal University, Jinan, China

**Keywords:** Attention, Cognitive control, Human behaviour

## Abstract

That younger individuals perceive the world as moving slower than adults is a familiar phenomenon. Yet, it remains an open question why that is. Using event segmentation theory, electroencephalogram (EEG) beamforming and nonlinear causal relationship estimation using artificial neural network methods, we studied neural activity while adolescent and adult participants segmented a movie. We show when participants were instructed to segment a movie into meaningful units, adolescents partitioned incoming information into fewer encapsulated segments or episodes of longer duration than adults. Importantly, directed communication between medial frontal and lower-level perceptual areas and between occipito-temporal regions in specific neural oscillation spectrums explained behavioral differences between groups. Overall, the study reveals that a different organization of directed communication between brain regions and inefficient transmission of information between brain regions are key to understand why younger people perceive the world as moving slow.

## Abstract (web summary)

An EEG study using event segmentation theory found adolescents segment movies into fewer, longer episodes than adults. Differences in neural communication patterns between brain regions explain why younger people perceive the world as moving slower.

## Introduction

That younger individuals perceive the world as moving slower than adults is a familiar phenomenon. Waiting for the next birthday to come seems “forever” for children, but the older we get, the faster “time flies”. Yet, it is an open question why that is. One possible answer relates to age-specific differences in the neural mechanisms underlying time and event perception. In this study, we relied on event segmentation theory (EST)^1,2^ to identify such differences. EST was developed to elucidate the principle underlying the way people segregate continuous incoming information in a given situation into meaningful units of coherent events, so-called event segments^3,4^. According to EST, this principle enables the human brain to understand what is happening now and to predict what might happen next. This process is guided by active working event models held in working memory, which can be considered as representation of the current situation according to EST and thus generate predictions of what might happen in the very near future. Whenever this prediction fails, an event boundary marking the end of an event segment and the beginning of a new event segment is set and the working event model is updated to reflect the new situation^2,5^. The working event model and its updating process are influenced by previously acquired knowledge about the usual course of events (i.e., event schemata)^2,6^. Thus, while interacting or structuring incoming information in a given situation, the maintenance of working event models in working memory is crucial^2,7^. These mechanisms might be important for understanding why younger individuals perceive the world as moving slower than adults.

Working memory capacity is known to increase during cognitive development^8–10^ and individual differences in this possibly affect how events are segmented and interpreted^3,11^. On average, children seem to be sufficiently able to segment events by the age of five, and their performance becomes more comparable to adults from then on^12–14^. Considering EST more closely, there is evidence that EST-related mechanisms differ between younger and older adults^15^, as well as between infants and adults^16^, but the neural mechanisms explaining these aspects are still elusive, particularly when it comes to the temporal dynamics of these processes. Yet, brain structures thought to be relevant for event segmentation dynamics, such as the medial frontal cortex and the dorsolateral prefrontal cortex^2,17–24^, undergo considerable changes between adolescence and young adulthood, which parallels increases in working memory capacity^25–29^. The working event model’s performance to predict what is coming up next is assumed to be evaluated by error detection mechanisms comparing the event model’s predictions to what actually happens^1,2,30,31^. These functions are supported by the dopaminergic system^32–34^, which continues to mature during adolescence as well^35–37^.

The purpose of the current study is to provide an in-depth analysis of the neurophysiological dynamics underlying differences in event segmentation between adolescents and (young) adults. Based on previous work of Prochnow et al.^38^, we combine different EEG analysis techniques to better understand the age-group-specific oscillatory brain activities and connectivity between different brain regions while watching natural scenes. These measures are considered particularly suitable to assess the temporal aspect of event perception. Moreover, oscillatory activity at different frequencies is assumed to reflect a fundamental principle of information processing^39,40^ that is of relevance for the questions of how information about extended events is integrated for behavioral control^41^. As mentioned above, the main mechanistic elements in EST are (currently maintained) event models and (stored) event schemata^2–4,42^. Working memory and the transition of information to a long-term "knowledge system" are functions related to alpha band activity (ABA; 8–12 Hz), which is assumed to be a constituent of an inhibitory gating mechanism^43–46^. Thus, ABA might be an essential mechanism for the comprehension and thus segmentation of events. ABA might differ between children/adolescents on the one hand and adults on the other, as adolescents might have fewer event schemata available, due to fewer experiences with various situations compared to adults. Given the significance of maintaining and updating working memory^42^, beta band activity (BBA; 15–30 Hz) is also likely to be relevant to consider. BBA is assumed to manage the top-down transfer of latent working memory content into working memory, while simultaneously representing the participants’ ongoing monitoring activities in relation to situational changes^47^. Another mechanistic element, closely related to the interplay of event models and event schemata, is a continuous prediction process that leads to closure of the current event model and the opening of a new one whenever the event model’s predictions become incorrect^2–4,42^. Theta band activity (TBA; 4–7 Hz) modulations have been linked to such prediction error signaling^47–49^. Given the developmental changes in the dopamine-associated error detection mechanism outlined above, the differences between adolescents and adults might thus also be reflected in the theta frequency band. Since it is the connectivity between brain regions that is prone to vast changes between children/adolescents and adults^50–54^, it is important to consider that particularly altered connectivity patterns and the transfer of information between brain regions within alpha, beta and theta band activities is central for mechanistic insights. This is also the case because recent theoretical considerations suggest that event segmentation and perception-action integration in general is mediated via distinct contributions of ABA, BBA and TBA in specific fronto-parietal networks^41^. To identify the connectivity patterns and their differences in detail, we examine linear and non-linear connectivity through nonlinear causal relationship estimation using artificial neural network (nCREANN)^55,56^. This approach offers the possibility to consider linear and non-linear connectivity patterns separately and to draw conclusions about the direction of this connectivity^55–57^. Likely, the adolescent and thus not fully matured brain shows a less well-organized communication (connection) between brain regions than the adult brain^50–54^ that explains differences in the behavioral patterns between groups and inter-individual variations.

## Results

### Adolescents compartmentalize incoming information into fewer encapsulated segments

**Fig. 1:**
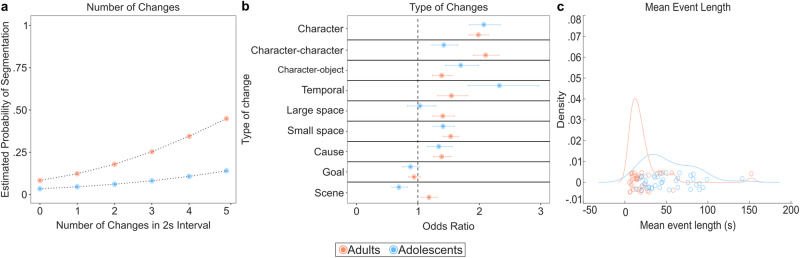
Behavioral results for adults and adolescents. Results of the mixed-effects logistic regression analyses and the mean event length based on a sample size of *n* = 40 for adult group and *n* = 39 for adolescents. Orange dots/lines denote the adult group, blue dots/lines denote the adolescent group. Figure part (**a**) shows the estimated probability of segmentation (*y*-axis) depending on the number of changes in an interval (*x*-axis) in adolescents and adults. Figure part (**b**) shows the odds ratios (dots) with their 95% confidence interval (error bars) indicating the likelihood of segmentation (*x*-axis) depending on the type of change (*y*-axis) for both groups. The factor has a significant effect on the segmentation probability if the confidence interval does not include 1 (dashed line). Figure part (**c**) shows the density (*y*-axis) of the mean event length in seconds (*x*-axis) separately for the groups.

Adolescent and adult participants conducted an event segmentation task while watching the movie “The Red Balloon”^58^. For both age groups, the number of situational changes was calculated for intervals of 2 s. Overall, there were 518 intervals with no changes, 278 intervals with only one change, 106 intervals with two changes, 52 intervals containing three changes, 29 intervals with four changes, and 4 intervals with five situational changes. The number of changes in an interval was used as predictor in a mixed-effects logistic regression analysis predicting the likelihood of a response in the event segmentation task, resulting in coefficients with *p*-values and Odds Ratios (OR) with Confidence Intervals (CI) as measures of significance. The mixed-effect logistic regression with significant intercept (−2.396, *p* < 0.001) revealed significant coefficients of the number of changes (0.438, *p* < 0.001; OR = 1.550, 95% CI = 1.513 − 1.588), group (−0.954, *p* < 0.001; OR = 0.385, 95% CI = 0.281 − 0.527) and, importantly, their interaction (−0.132, *p* < 0.001; OR = 0.876, 95% CI = 0.840 − 0.914). As it is shown in Fig. 1a, these results indicated that the probability of segmentation increased as the number of situational changes increased for both subject groups while the slope of this increase was flatter for the adolescent group (Intercept: −3.342, *p* < 0.001 Coefficient: 0.306, *p* < 0.001) compared to the adult group (Intercept: −2.399, *p* < 0.001, Coefficient: 0.438, *p* < 0.001). A comparison of the coefficients of the two groups using a *z*-test revealed a significant group difference (*z* = 5.821, *p* < 0.001).

Subsequently, we ran a second mixed-effect logistic regression to estimate the relationship between each of the nine types of situational change with event segmentation probability for both samples. In this analysis, the multicollinearity was not a concern since all variance inflation factors (VIF) were below 5 (VIF ≤ 4.01). The ORs of all nine types of situational changes and the ORs of their interaction with the predictor group are presented in Table 1.

**Table 1:** Odds ratio (OR) for Type and the Interaction of Type and Group

Situational change type	OR Type (95% CI)	OR Type × Group (95% CI)
Character	**1.995** (1.845-2.157)	1.024 (0.888-1.182)
Character-character	**2.114** (1.915-2.333)	**0.666** (0.558-0.795)
Character-object	**1.394** (1.242-1.566)	1.204 (0.990-1.464)
Small-space	**1.541** (1.421-1.671)	0.902 (0.779-1.046)
Large-space	**1.405** (1.239-1.593)	**0.736** (0.571-0.949)
Temporal	**1.547** (1.319-1.815)	**1.505** (1.126-2.013)
Cause	**1.389** (1.255-1.537)	0.963 (0.802-1.157)
Goal	0.938 (0.847-1.039)	0.935 (0.776-1.127)
Scene	**1.187** (1.067-1.320)	**0.575** (0.467-0.709)

*Note*. The 95% Confidence Interval (CI) is given in brackets. Significant OR are displayed in bold font.

The results of the logistic regression model considering situational change type and group and interactions of these predictors are displayed in Fig. 1b. Significant interactions of situational change type and group were only present for character-character, temporal, large-space and scene changes. In Fig. 1b, significance of a predictor can be seen by the CIs of the depicted ORs that are not including 1 (indicated by the vertical dotted line), while an interaction of change type and group can be deemed significant when the CIs of the groups do not overlap. In the adult group, the Character-character change was better able to predict the segmentation than in the adolescent group (adolescents: OR = 1.422, 95% CI = 1.227–1.647; adults: OR = 2.102, 95% CI = 1.905 − 2.320). Moreover, large-space changes were predictive of segmentation in adults (OR = 1.404, 95% CI = 1.239 − 1.593) but not in adolescents (OR = 1.033, 95% CI = 0.829 − 1.289). In contrast, temporal changes were less predictive of segmentation in the adults (OR = 1.546, 95% CI = 1.318 − 1.813) than in adolescents (OR = 2.326, 95% CI = 1.825 − 2.966). Regarding the scene changes, a scene change increased the segmentation probability in the adult group (OR = 1.181, 95% CI = 1.062 − 1.313), but decreased the segmentation probability in the adolescent group (OR = 0.690, 95% CI = 0.576 − 0.826).

The comparison of the average segment length between the two groups showed a difference in the mean duration of the indicated segments (t(77) = 4.92, *p* < 0.001; Fig. 1c).

Both adolescents and adults were asked how well they understood the plot of the movie and how well they understood the task in questionnaires after they conducted the task. The groups did not differ in their understanding of the plot (*z* = 0.48, *p* = 0.630) and in their understanding of the task (*z* = 1.07, *p* = 0.282).

### Oscillatory architecture of event segmentation in adults and adolescents

**Fig. 2:**
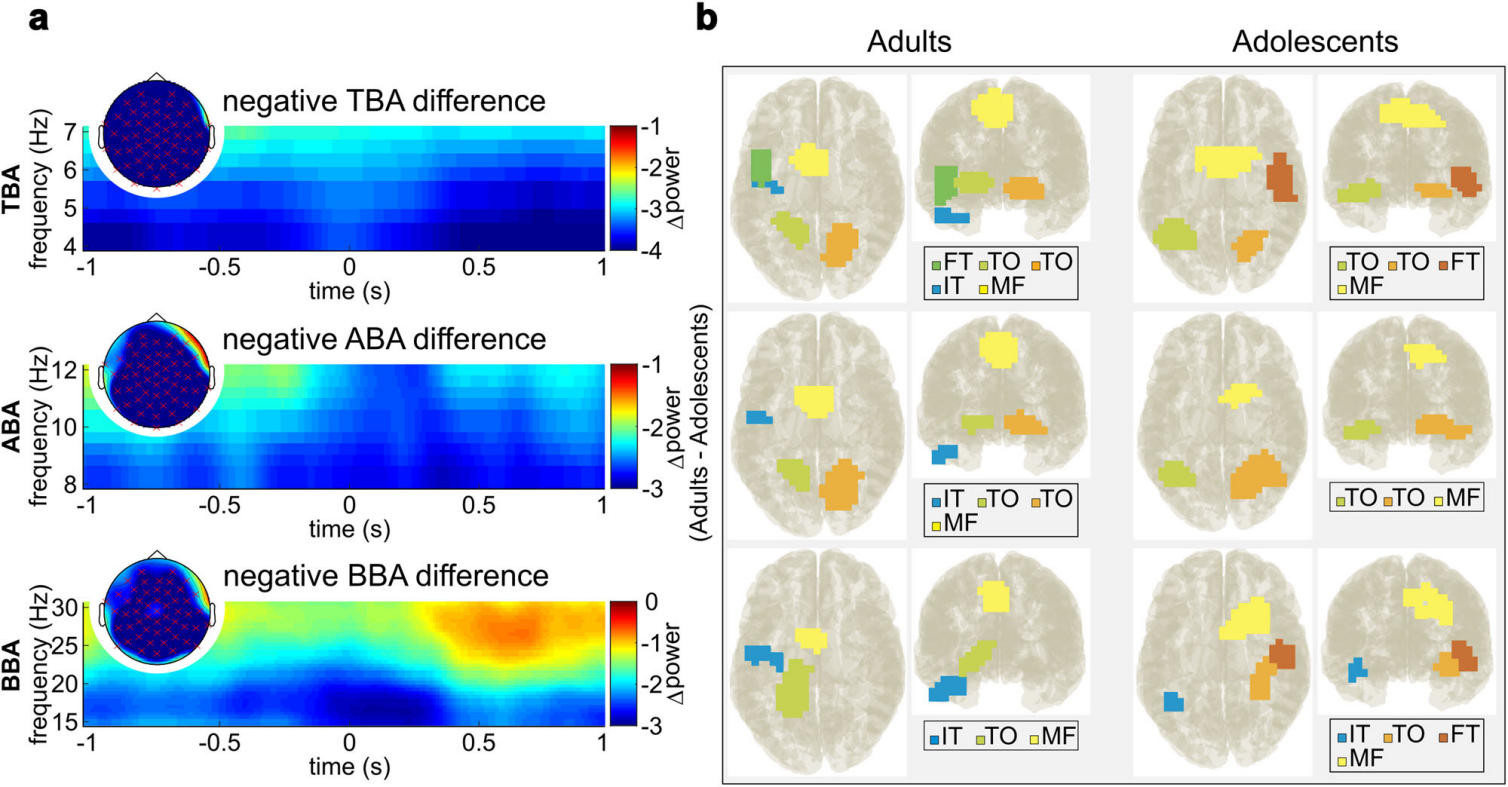
TF and DBSCAN results for adults and adolescents. **a** Results of the time-frequency (TF) decomposition (TF plots) and the cluster-based permutation testing (topographic plots). TF plots display the mean power difference for each frequency band between the Adult and Adolescent groups across the significant electrodes identified through cluster-based permutation testing. Topographic plots illustrate the average power difference for each frequency band between the Adult and Adolescent groups within the significant time windows identified through cluster-based permutation testing. **b** The outcomes of the DBSCAN algorithm for adolescents and adults are presented. The first two columns display the top and back views for the Adults, while the last two columns depict the top and back views for the Adolescents. IT inferior temporal cortex, FT fronto-temporal cortex, TO temporo-occipital cortex, MF medial frontal cortex, details regarding the involved Brodmann areas can be found in the main text.

The above analysis revealed that there are differences between adolescents and adults in event segmentation; that is in the probability to set a segment boundary based on information presented in the movie. For the neurophysiological data analysis, we, therefore, focused the analysis on Boundary intervals (BIs). For these segments, the cluster-based permutation test revealed a negative cluster (*p* < 0.001) for TBA (adults < adolescents) as shown in Fig. 2a. This negative cluster shows that adolescents revealed higher TBA at frontal, parietal, temporal, and occipital electrodes from −1s to 1s relative to the button press. Another cluster-based permutation test also revealed a negative cluster (*p* < 0.001) for the ABA in Boundary intervals (BIs) (adults < adolescents) as shown in Fig. 2a at frontal, parietal, temporal, and occipital electrodes from −1s to 1s relative to the button press as well. Furthermore, a third cluster-based permutation test again revealed a negative cluster (*p* < 0.001) for the BBA (adults < adolescents) as represented in Fig. 2a at frontal, parietal, temporal, and occipital electrodes from −1s to 1s relative to the button press.

The Dynamic Imaging of Coherent Sources (DICS) beamforming and applying the Density-Based Spatial Clustering of Applications with Noise (DBSCAN) algorithm on the results of the DICS beamforming, in order to localize the generators of the activity of the three frequency bands of interest (TBA, ABA and BBA) within the brain. The results revealed the following clusters of activity based on the Neural Activity Index (NAI), which were different between adults and adolescents: Regarding TBA, the adults revealed activities in medial frontal (MF; BA6/BA8/BA9/BA24), left fronto-temporal (l-FT; BA16/BA21/BA22/BA44), left temporo-occipital (l-TO; BA19/BA36/BA37), left inferior temporal (l-IT; BA20) and right temporo-occipital (r-TO; BA19/BA37) regions (Fig. 2b). In the adolescent group, activity was evident in medial frontal (MF; BA6/BA8/BA9/BA24), left temporo-occipital (l-TO; BA19/BA37), right fronto-temporal (r-FT; BA6/BA16/BA21/BA22/BA41), and right temporo-occipital (r-TO; BA19/BA37) regions (Fig. 2b). Regarding ABA, and in the adult group, there were four clusters of activity encompassing the medial frontal (MF; BA6/BA8/BA24), left temporo-occipital (l-TO; BA19/BA37), and left inferior temporal (l-IT; BA20) and right temporo-occipital areas (r-TO; BA19/BA37). In the adolescent group activities were found in medial frontal (MF; BA6/BA8/BA9/BA24), and bilateral temporo-occipital (l-TO and r-TO; BA19/BA37) regions (Fig. 2b). With regards to BBA, the adult group revealed activities in the medial frontal (MF; BA6/BA8/BA24), left inferior temporal (l-IT; BA20), and left temporo-occipital (l-TO; BA19/BA36/BA37) areas; in the adolescent group activities were found in the medial frontal (MF; BA6/BA8/BA9/BA24/BA44/BA45), left inferior temporal (l-IT; BA37), right fronto-temporal (r-FT; BA36/BA37), and right temporo-occipital (r-TO; BA16/BA21/BA22) areas (Fig. 2b).The DICS beamforming and DBSCAN algorithm were also run for No-Boundary intervals (NBIs), showing that the brain regions obtained for the Boundary intervals (BIs) are specific to them (Supplemental Fig. 2).

### Distinct patterns of directed and bidirectional connectivity between groups

**Fig. 3:**
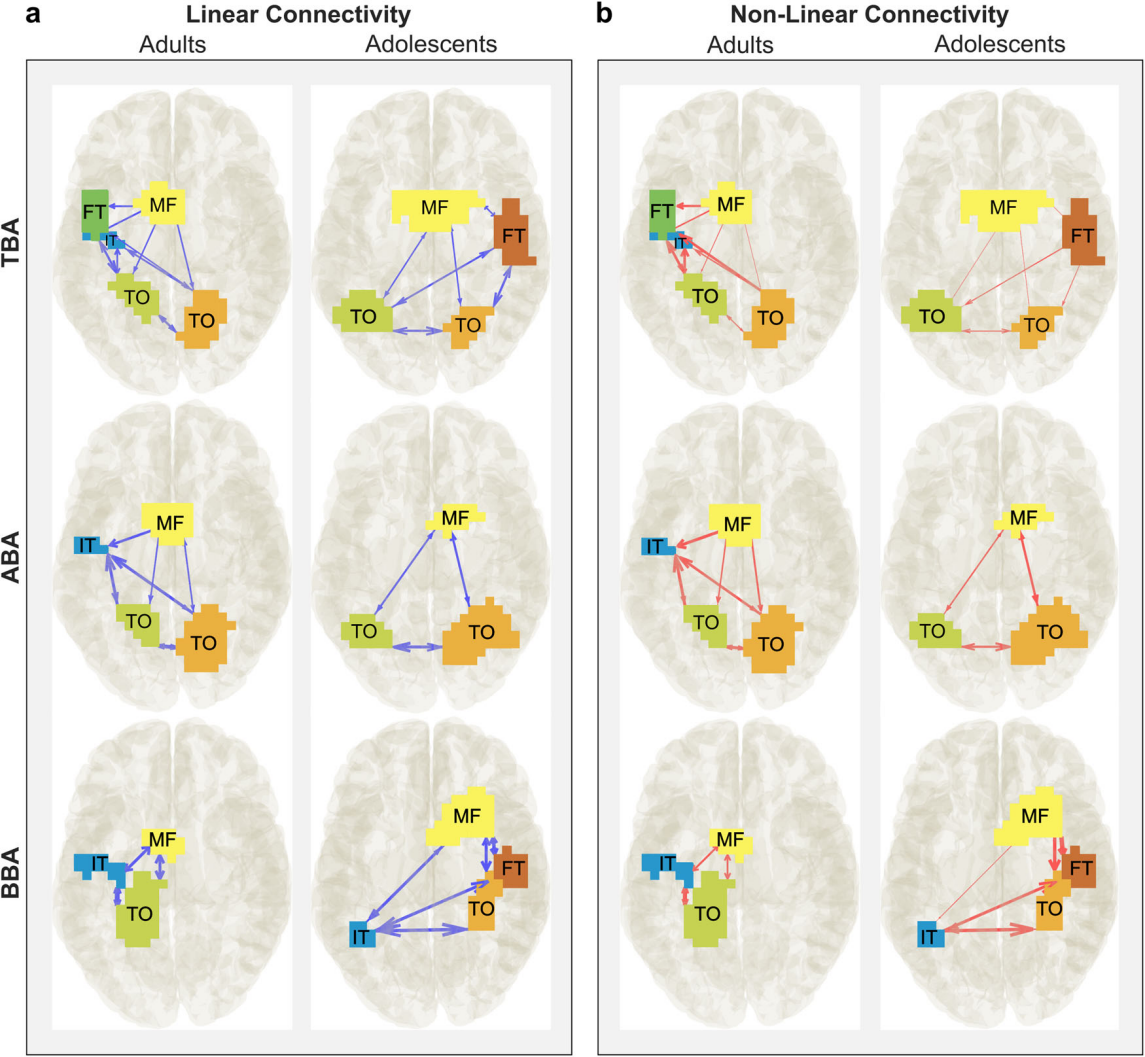
nCREANN connectivity results for adults and adolescents for BI. The nCREANN Linear and Non-Linear connectivity between brain regions for each frequency band and each group after surrogate testing is displayed. This means that the significant connections (those that exceeded 90% of the surrogate data) were averaged across subjects in each group for Linear and Non-Linear connectivity. The presented view is top view. The blue arrows in figure part (**a**) represent Linear connectivity and the red arrows in figure part (**b**) represent Non-Linear connectivity. The thickness of the arrows and the arrowheads indicates the strength of connectivity. IT inferior temporal cortex, FT fronto-temporal cortex, TO temporo-occipital cortex, MF medial frontal cortex, details regarding the involved Brodmann areas can be found in the main text.

The significant connections (those that are stronger than 90% of the surrogate data) were averaged across subjects in each group. The results of the connectivity analyses are shown in Fig. 3. For a better interpretability of the connectivity parameters, these were scaled by multiplying these by the factor 100. The linear and non-linear connectivity patterns are presented separately.

### Linear connectivity

The linear connectivity pattern after surrogate testing for adult and adolescent groups in each frequency band is shown in Fig. 3a. The exact values for each frequency band and group can be found in the Supplemental Tables 1–6.

In the theta band of the adult group, there is a linear effect from MF to the l-IT (2.356), and from l-TO to l-IT clusters (2.337). Furthermore, bidirectional linear connectivity between l-FT / l-IT (4.130 / 3.513), l-FT / l-TO (2.813 / 2.502), and l-TO / r-TO (2.396 / 2.310) is observable in Fig. 3a. In the adolescent group, there is a strong linear connection from l-TO to r-FT (2.294), and bidirectional connectivity between l-TO / r-TO (2.846 / 2.535) and r-TO / r-FT (2.455 / 2.763) clusters.

In the alpha frequency band, there are both different and similar patterns in the adult and adolescent groups. In both groups, there are dominant bidirectional linear connections between right temporo-occipital and left temporo-occipital regions (adults: 4.797 / 4.694, adolescents: 3.834 / 2.684). In adults, l-IT is significantly influenced by the MF (3.246), r-TO (3.464), and l-TO (3.420), but in adolescents, there is a connection from r-TO to MF (2.994), and from MF to the l-TO (2.326).

In the beta frequency band, there is strong bidirectional linear connectivity between active regions in adults (l-IT / l-TO (5.699 / 6.029), l-IT / MF (3.633 / 3.310), MF / l-TO (3.335 / 3.814)) and in adolescents (l-IT / r-TO (4.194 / 4.043), l-IT / r-FT (3.735 / 3.666), l-IT / MF (2.471 / 3.780), MF / r-TO (3.905 / 3.157), MF / r-FT (4.436 / 3.936), r-FT / r-TO (10.812 / 11.517)).

The linear connectivity values (without the surrogate testing) were averaged across subjects in each group and shown in the Supplemental Tables 13–18, and shown in the Supplemental Fig. 1.

### Nonlinear connectivity

Figure 3b shows the nonlinear connectivity after surrogate testing for adult and adolescent groups in each frequency band. The exact values for each frequency band and group can be found in the Supplemental Tables 7–12.

In the theta band of the adult group, I-FT is influenced by nonlinear effects of all other regions (MF (2.409), r-TO (2.802), l-TO (3.570), and l-IT (4.987)). A similar pattern is seen for the l-IT (r-TO (2.230), l-TO (3.521), and l-FT (4.932)), however, it is less affected by MF (1.579). The adolescent group have very weak nonlinear connectivity in the theta band.

Furthermore, in the alpha frequency band in adults, MF has a nonlinear influence on l-IT (3.623), r-TO (2.021), and l-TO (1.985), but it is not strongly affected by the other regions. There are also bidirectional nonlinear connectivity values between r-TO / l-TO (3.923 / 2.993), l-TO / l-IT (3.902 / 1.870), and r-TO / l-IT (2.981 / 1.871) clusters. In adolescents, there are bidirectional connections between all active regions (r-TO / l-TO (2.260 / 2.747), r-TO / MF (2.685 / 2.847), and l-TO / MF (1.562 / 2.105)).

In the beta frequency band, nonlinear connectivity is observed in both groups. In adults, there are strong bidirectional connections between all active regions (MF / l-IT (2.403 / 2.298), l-TO / l-IT (5.784 / 4.401), and l-TO / MF (2.574 / 2.647)). In adolescents, r-FT (5.449) and r-TO (4.310) are influenced by MF, and r-TO (2.020) nonlinearly affects l-TO as well. The strongest nonlinear connection is between r-FT and r-TO (10.450 / 9.724). The non-linear connectivities (without the surrogate testing) were averaged across subjects in each group and shown in the Supplemental Tables 19–24, and shown in the Supplemental Fig. 1.

The connectivity analyses of linear and nonlinear connectivity were also run for No-Boundary intervals (NBIs), showing that the brain regions obtained for the Boundary intervals (BIs) are specific to them (values could be seen in Supplemental Tables 38–49 and the figure could be found in the Supplemental Fig. 3).

### Connectivity patterns explain behavioral variation in event segmentation

Only significant correlations between the behavioral segmentation probability and above-average connectivities will be reported. The exact results regarding the partial correlation analysis between all of the connectivities in all three frequency bands and the behavioral segmentation probability can be found in the Supplemental Tables 25–36 (TBA: Supplemental Tables 25–28; ABA: Supplemental Tables 29–32; BBA: Supplemental Table 33–36).

Regarding TBA, none of the connectivities showed a significant correlation with the behavioral outcome in neither of the groups (|r| ≤ −0.09, *p* ≥ 0.981).

With respect to ABA, in the adult group, there were significant correlations between the behavioral segmentation probability and the linear connectivities from MF to l-TO (*r* = −0.50, *q* = 0.050) and from l-TO to MF (*r* = 0.48, *q* = 0.050).

With regard to BBA, there were no significant correlations between the behavioral outcome and any of the connectivities in neither of the groups (|r| ≤ −0.09, *p* ≥ 0.974).

## Discussion

In the current study, we examined the neural mechanisms underlying event segmentation as suggested by Event Segmentation Theory (EST)^1,2^ to investigate the neurophysiological mechanisms underlying the observation that adolescents perceive the world as moving slower than adults.

When segmenting a movie into meaningful units, adolescents and adults performed differently. Adolescents were less likely than adults to set an event boundary (i.e., mark consecutive frames in the film as not belonging to each other) when the number of situational changes in the movie increased, suggesting that adolescents seem to act on longer time scales, just like younger children^16^. According to EST, a segment boundary is set whenever the actually obtained information violates the individual/subjective prediction. For adolescents, it was particularly situations when there was a temporal jump between two consecutive frames (i.e., temporal changes) which were most important to set a segment boundary. For adults, it was rather the change in interaction of characters in consecutive frames of the movie, e.g., when characters met or ended a conversation, that was crucial to set an event boundary. This pattern already shows that different aspects of information are being used to structure ongoing events into discrete segments useful for behavioral organization.

The neurophysiological data provide more insights into the likely cause of difference between both age groups. BBA was observed in medial frontal and temporal brain areas. The activity in the medial frontal cortex might reflect the updating of the working event model, one of the processes relevant to set an event boundary according to EST^42^. Of note, the localization of BBA included a long time period around the response indicating an event boundary, whereas motor preparatory processes are only evident for a very short period of time right before the response^38^. This makes it unlikely that medial frontal activity reflects motor-related processes. BBA in temporal and occipitotemporal cortex, on the other hand, might reflect top-down processing in the visual domain when participants undergo an attention-related process^59–61^. For the adults, the most activated brain regions are mainly located in the left hemisphere, whereas for the adolescents, the most activated brain regions are distributed across both hemispheres. Upon examining the connectivity findings between these regions with the largest BBA, the connectivities are bidirectional between the medial frontal, occipitotemporal and temporal brain areas in both adults and adolescents without marked differences between linear and non-linear connectivity. However, considering the distribution of involved brain regions in both age groups, the results suggest that there might be less sufficient updating process coordination in adolescents than in adults. This interpretation is further corroborated by the findings in TBA.

According to EST, the updating of a working event model and the establishment of an event boundary happens when the prediction of the current working event model fails^2,42^. TBA reflects predictive coding and processing^47–49^, suggesting that the observed increased TBA in the medial frontal cortex during event segmentation for adults compared to adolescents may reflect the failed prediction of the event model causing the event segment to be closed. However, TBA has also been related to attentional sampling^62,63^ and input integration^64,65^, which might be reflected in TBA in temporal and occipitotemporal brain areas in the current study. These ventral stream^66–68^ regions process the “what information”, which is the information central to decide whether to close an event segment or not. Crucially, there was a distinct pattern of TBA connectivity between the two age groups with respect to the strongest connectivities: in adults, there was a considerable directed effect from the medial frontal cortex and occipitotemporal areas to frontotemporal and inferior temporal regions showing a top-down control effect from the medial frontal cortex to areas of the ventral stream around the time an event boundary is set. In addition, occipitotemporal regions showed bidirectional connectivities with the temporal areas, suggesting close communication between different areas of the visual system around the time of an event boundary. In contrast, in adolescents, the medial frontal cortex did not show strong connectivities to other regions and thus did probably not exert a strong directed effect upon ventral stream visual association cortices. This suggests that in adolescents, the integration and identification of the visual input from the movie is less top-down controlled than in adults, leading to a lower probability to identify and thus to set an event boundary. Of note, also in TBA, linear and non-linear connectivity showed no marked differences. While these findings already point to a relevant mechanism, there was an even stronger relationship established between connectivity patterns and segmentation behavior when the alpha band activity is concerned.

The medial frontal cortex ABA might reflect top-down control of the access to information in working memory through inhibitory gating^43–46,69,70^. ABA is assumed to reflect an inhibitory gating of access to episodic memory information^71^, which exhibits conceptual parallels to event schemata, one of the core elements of EST^3,4,19^. ABA potentially reflects the control of access of incoming information to an “event schema storage”. ABA was also observed in ventral stream occipitotemporal regions. There, ABA might reflect the inhibitory gating of sensory information by suppressing irrelevant information and thus emphasizing relevant information to decide when to close an event segment. Since the behavioral data clearly shows that adults and adolescents use different aspects in incoming information (i.e., for adolescents: observed jumps in time; for adults: change in interaction of characters) it is reasonable that ventral stream areas encoding the “what information” play a role. In adolescents, this modulation is evident in rather lower-level areas of the visual stream, whereas in adults more down-stream, higher-level parts of the ventral stream encompassing the inferior temporal cortex were also activated. Crucially, when considering the connectivity between the most activated regions within the ABA, differences between the age groups emerged, similarly in linear and non-linear connectivity: while the strongest directed connectivities converge from the medial frontal and occipitotemporal regions to the temporal cortex in adults, there is no such a convergence towards one of the brain regions in adolescents. Thus, the communication between top-down-controlling areas and the ventral stream which is related to the processing of visual information appears to be closer and thus better organized and more efficient in adults compared with adolescents. This connectivity pattern explained inter-individual variations in behavioral segmentation probability in adults, but not in adolescents. The stronger the directed top-down control connectivities towards visual cortices (i.e., temporooccipital regions) in the alpha frequency band were, the less likely it was that an event boundary was set based on the number of situational changes. However, the stronger the vice-versa directed connectivity from visual cortices to the top-down-controlling medial frontal cortex was, the more likely it was that an event boundary was set based on the number of situational changes. Therefore, directed inhibitory gating exerted by the medial frontal cortex on the ventral processing stream and vice versa reflects the mechanistic elements explaining behavior during event segmentation. The lack of correlations in the adolescent group may emerge because adolescents have fewer event schemata due to fewer experiences with different situations, i.e., a limited understanding of how events usually evolve and how one event builds upon another. Therefore, it is less likely to suppress what is to be considered irrelevant by ABA in adolescents.

In summary, the current study examined the conundrum why younger individuals perceive the world as moving slower than adults. Based on the Event Segmentation Theory (EST), we identified neural processes explaining this phenomenon. Adolescents partitioned incoming information into fewer encapsulated segments or episodes of longer duration than adults, which was reflected in differences in theta, alpha and beta band activity. Especially alpha band activity is mechanistically relevant, and likely reflects the access to event schema storage in occipitotemporal and inferior temporal regions during event segmentation. Adolescents show modulation in lower-level visual areas, while adults engage higher-level cortical regions. Connectivity differences emerge, with adults exhibiting directed convergence from medial frontal and occipitotemporal regions to the temporal cortex directly predicting the probability of event segmentation. This was not the case in adolescents, where medial frontal regions did not exert directed top-down control on lower-level regions.

## Methods

### Participants

The study compared 45 healthy adults (aged 18–30 years) and 45 healthy adolescents (aged 11–16 years). 5 adults and 6 adolescents were excluded from the analysis due to too few responses during the task, issues with the EEG data, and/or reporting a psychiatric disorder. Therefore, the final number of participants for the adult sample was *N* = 40 (20 males, 25.65 ± 2.85 years), and for the adolescent sample *N* = 39 (15 males, 14.03 ± 1.60 years). An in-house database as well as advertisements were used for recruiting participants. Adult participants were subjected to screening questions prior to and during participation and any neurological or psychiatric disorder, chronic or acute medication, and a history of substance abuse or dependence were excluded. Also, the parents or adult caregivers of the adolescents provided information about any physical or mental health diagnoses and mental health symptoms using an online questionnaire on *SoSci survey*^72^. All participants had normal or corrected-to-normal vision. The adolescents, their legal guardians, and adult participants were informed of the experiment’s protocol at the time of the appointment and provided their informed consent. All participants were financially reimbursed for their participation. The study was approved by the local ethics committee of the Medical Faculty of TU Dresden. All ethical regulations relevant to human research participants were followed.

### Task description

All participants performed an event segmentation task, which required them to press the space button with their right index finger in order to parse a movie into subjectively meaningful units. The short movie “The Red Balloon”^58^ with a duration of about 34 min was shown to the participants while their neurophysiological brain activity was recorded through EEG measurement. This movie was selected because it contains only a small amount of spoken language, frequent situational changes, and only short temporal jumps with preserved chronological order^2,4,20^. Before the participants watched the movie, adolescents were presented with a video clip of a man decorating a room for a party (duration of ~6 min)^4^ and adults with a video clip of a man assembling “Duplo” construction blocks (duration of ~2:30 min)^4^ for practice. The movie was cut into four 7–10 min clips with pauses in between each clip for the adults in accordance to Zacks et al.^4^. For the adolescent sample, the movie was cut into three clips of about 10 min duration with pauses in between with the aim to make the potentially exhausting task seem shorter for this sample by showing less clips, and thus fostering engagement and completion of the task. All participants were free to continue their task by pressing the space button after a break of self-chosen length between the clips. All videos were shown using the software “Presentation” (NeuroBehavioral Systems Inc.). Thus, the setup was similar to a previous study by Prochnow et al.^38^.

### Experimental design and statistical analyses

In a previous study by Zacks et al.^4^, nine different types of situational changes have been specified in the movie “The Red Balloon” and were coded frame by frame. The situational change types are as follows: (i) “Temporal changes” were defined when the frame following a cut was temporally detached from a frame before the cut. “Spatial changes” were divided into (ii) “Large space changes”, when the character’s location in two successive frames has changed, and (iii) “Small space changes” were specified when there were any locational changes in the camera’s point of view. (iv) “Character changes” were coded at the time that the focus of an action or behavior in a scene was a character or an animate character, and this focus was different from the ones in the preceding frame. (v) “Character-character changes” referred to the time the characters’ actions count as interaction with each other’s, such as moving or running toward each other, conversing, gesturing, or having physical contact. (vi) “Character-object changes” were coded when the dynamic between the objects and character changed or the character began to use the object in a different way compared to the previous frame. (vii) “Cause changes” were identified when the reasoning underlying the actions of the present frame was not a result of something that was seen in the previous one. (viii) “Goal changes” were coded as the time that a character’s behavior associated with a goal was different from the one in the preceding frame and (ix) “Scene changes”, which were defined as moments when the entire shot was replaced with a new one.

Referring to Zacks et al.^4^, each clip in the movie was split into intervals with 2s duration (982 intervals in total) for the behavioral analysis. Boundary intervals (BIs) were assigned to the intervals that contained an observer’s key press while intervals without such a behavior were labeled as No-Boundary intervals (NBIs). The situational changes defined by Zacks et al.^4^. were counted for each interval regardless of the change type, and it was coded for each change type for each interval if this change type occurred or not. To statistically analyze the behavioral data, mixed-effects logistic regression (R version 4.2.1, ‘glmer’ function) was used to estimate the segmentation probability (criterion) as the function of the number of changes and group as the two predictors. We constructed two mixed-effects regressions models for distinct objectives: (i) predicting segmentation probability from the number of changes for each sample (i.e., adolescents and adults), and (ii) to evaluate the relationship between each type of change and segmentation pattern for both groups. For both models, the random intercept for subjects was estimated to account for the variability between subjects, and odds ratios were calculated based on the coefficient results of fixed effect to be able to compare the influence of the different predictors. For (i), the number of changes irrespective of the type of change was counted for each 2s interval and fed into the regression analysis as one predictor (ranging from 0 to 5) and the other predictor was the group (adolescent [0] vs adults [1]). The outcome was if there was a response by the participant indicating an event boundary within that interval or not (i.e., BI [1] vs. NBI [0]) for each group. For (ii), nine predictor variables were the presence [1] or absence [0] of situational change of each of the 9 situational change types within a 2s interval (see above), the tenth predictor was group (adolescent [0] vs adults [1]) and the outcome variable was the same as in (i). The variance inflation factor (VIF, R version 4.2.1, ‘vif’ function) was calculated to check for multicollinearity between the predictors. In addition to the logistic regression models, an independent *t*-test was used to compare between groups the mean length of the segments defined by the participants.

### Task-related questionnaires

Both groups were asked to indicate how well they understood the plot of the movie and how well they understood the task in a questionnaire given to them right after conducting the event segmentation task. The questionnaire consisted of statements that should be rated by the participants on a 5-point Likert scale (1—strongly agree, 5—strongly disagree). The responses were compared using a rank sum test as implemented in MATLAB. *N* = 2 adult participants and *N* = 3 adolescent participants did not fill in the questionnaire.

### EEG recording and pre-processing

The EEG signals of 60 equidistantly positioned Ag/AgCl electrodes in an elastic cap (EasyCap Inc.) (reference electrode at Fpz, ground electrode at θ = 58, *ϕ* = 78) were recorded using a BrainAmp amplifier (Brain Products Inc.) while participants were watching the movie. The electrode impedances were kept under 5 kΩ. An offline down-sampling process was done from the primary sampling rate of 500 Hz to 300 Hz. EEG pre-processing was conducted by using the “Automagic” pipeline^73^ and running EEGLAB^74^ on MATLAB 2019a (The MathWorks Corp), as done in previous studies^75–77^. As the first steps of the pre-processing, flat channels were removed and the EEG data were re-referenced to an average reference, followed by the implementation of the PREP preparation pipeline^78^ and the EEGLAB “clean rawdata()” pipeline, respectively. PREP uses a multitaper algorithm to remove line noise at 50 Hz and then adds a robust average reference after removing contamination by bad channels. Afterward, the “clean rawdata()” pipeline used a 0.5 Hz Finite Impulse Response (FIR) high-pass filter (order 1286, stop-band attenuation −80 dB, transition band 0.25 − 0.75 Hz) to detrend the EEG data. Noisy or flat-lined channels as well as outliers were identified and eliminated. In order to reconstruct epochs with abnormally strong power (>15 standard deviations relative to calibration data) in the partitioned data (see below), Artifact Subspace Reconstruction was used (ASR; burst criterion: 15^79^;). Time periods that could not be recreated were eliminated, followed by the application of a low-pass filter of 40 Hz (sinc FIR filter; order: 86^80^;).

To remove the EOG artifacts, a subtraction method was applied^81^. The rest of the artifacts such as muscle, heart, and remaining eye artifacts were automatically organized and removed by using an Independent component analysis (ICA) based Multiple Artifact Rejection Algorithm (MARA^82,83^). To interpolate the absent and removed channels, a spherical method was used. The neurophysiological data were segmented into intervals of 2 s locked to the responses indicating an event boundary using the FieldTrip Toolbox (Oostenveld et al., 2011). To this end, the data were first separated into pieces of 4 s with a 50% overlap (2s). Subsequently, the central 2s of interest could be computed after eliminating 1s each of the data at the beginning and end of the 4s intervals. This procedure is important to avoid the edge effects in the time-frequency decomposition. The final analyses contained the data from –1s to 1s relative to the respective marker (i.e., response marker). The segments were allowed to overlap.

### Time-frequency decomposition and cluster-based permutation testing

We employed time-frequency (TF) decomposition by the application of the Morlet wavelets in the frequency domain for a between-subject design between the adult and the adolescent groups. The selected frequencies ranged between 3 and 30 Hz. The wavelet length in implicit Gaussian kernel standard deviations was three, with the number of cycles being linearly selected from three (for 3 Hz) to twelve (for 30 Hz). This procedure resulted in the estimation, at each time point and for each EEG sensor, of the average power in each of the three relevant frequency bands: theta (4–7 Hz), alpha (8–12 Hz), and beta (15–30 Hz). After that, a cluster-based permutation test as implemented in FieldTrip^84^ was computed for the time-frequency results of the three frequency bands of interest to compare the differences of the Boundary intervals (BIs) between the groups (i.e., adults vs adolescents). If a sample’s *t*-value was significant (*p* < 0.050) in the paired-sample *t*-test and this applied to at least two adjacent samples (such as two neighboring EEG channels or two subsequent time points), those samples were defined as a cluster. By using the Monte Carlo approach^85^, 1000 random draws were used to approximate the reference distribution of the permutation test. If the matching *p*-values were less than the critical alpha level of *p* = 0.0025, a cluster was deemed significant. Of note, cluster-based permutation testing is limited to the sensor-level analysis due to methodological reasons.

### Beamforming analyses

In order to assess the source activity of the frequency bands of interest (ABA, BBA, and TBA) within each of the groups, dynamic imaging of coherent sources (DICS) beamforming^86^ was conducted to localize their activations in brain regions, as done in previous studies by our group^87,88^. To compute the DICS beamforming, common spatial filters within each group were calculated from the cross-spectral density of a Fast Fourier Transformation (FFT) on the averaged theta (4–7 Hz), alpha (8–12 Hz), and beta (15–30 Hz) frequency bands. The DICS beamformer technique was utilized to project the location of activity onto a grid with an even spacing of 0.5 cm. This grid was created using the FieldTrip toolbox’s forward model template, which is based on the standard Montreal Neurological Institute (MNI) space and used as the source space. The sensor position calculation was provided from the spatial arrangement of electrodes in relation to the brain. Based on the geometrical and conductive properties of the head, the head model was constructed. Specifically, the head model employed is a Boundary Element Method (BEM) volume conduction model of the head^89^. In order to account for the increased noise with increasing distance from the sensors, i.e., towards the center of the head, the Neural Activity Index (NAI) was computed. To this end, the estimates of the source activity were divided by the corresponding estimates of local noise per voxel^90^. The power values were normalized using a decibel conversion in each interval. To identify the clusters with the largest activity in the frequency bands of interest within functional neuroanatomical regions for each group in the DICS-beamformed data, the Density-Based Spatial Clustering of Applications with Noise (DBSCAN) algorithm^91–93^ was applied as implemented in MATLAB. For this purpose, the threshold for the largest activity was set to the top 3% of the power distribution within regions labeled within the Automatic Anatomical Labeling (AAL) atlas^94^. In this way, the DBSCAN algorithm was used to identify neighboring voxels, with an epsilon of once the grid’s edge length and a minimum cluster size of two voxels. The results of the DBSCAN analysis were further restricted to functionally meaningful clusters by manually inspecting the size and/or associated AAL atlas labels.

### Effective connectivity analysis

The effective connectivity patterns in alpha, beta, and theta frequency bands between the brain regions (clusters) established by DICS beamforming and the DBSCAN algorithm were evaluated using the nCREANN (nonlinear Causal Relationship Estimation by Artificial Neural Network) method^55,56^. The nCREANN is a multivariate approach that utilizes artificial neural network (ANN) to estimate effective connectivity among multiple regions. The nCREANN is based on a nonlinear Multivariate Autoregressive (MVAR) model. In an MVAR model, the current samples of the brain regions are generated based on the interactions of the previous regions’ activities. The MVAR model is usually used to conceptualize temporal causality, where the cause affects the future. Unlike the conventional linear methods that mainly focus on linear MVAR models, the nCREANN method captures both linear and nonlinear dynamics of the information flow among cortical regions. For the organization of information transfer between cortical regions, it is relevant that interactions are highly nonlinear^56,95,96^, but the linear methods may oversimplify the complex brain function due to not considering nonlinear dynamics.

Furthermore, nCREANN has the ability to distinguish between linear and nonlinear causal relationships. Several lines of evidence suggest that linear and nonlinear principles are relevant for a better understanding of neuro-dynamics at a macroscale—that is between brain regions involved in various brain functions^97–99^. In the nCREANN method, the signals are modeled as a nonlinear MVAR process:

1 $$x\left(n\right)=f({x}_{p})+\sigma (n)$$

where ${x}_{p}={\left[{x}_{1}\left(n-1\right),{x}_{2}\left(n-1\right),\cdots ,{x}_{M}\left(n-p\right)\right]}^{T}\,$ is the vector of $p$ previous samples of (M) time series, and $\sigma \left(n\right)=\,{\left[{\sigma }_{1},{\sigma }_{2},\ldots ,{\sigma }_{M}\right]}^{T}$ is the model residual. This model is implemented by a single-hidden-layer feed-forward network. During the training, the subsequent samples, as the network’s outputs, are predicted based on previous samples of all signals. With nonlinear activation functions for hidden neurons and linear ones at the output layer, $f\left(.\right)\,$ is a nonlinear function that expresses how the $p$ previous samples cause the present values (Eq.(1)). This produces a nonlinear MVAR model which information about linear and nonlinear interactions is embedded in the network’s parameters^55,56^.

To implement this nonlinear MVAR model, a multilayer perceptron neural network with single hidden layer and 10 hidden neurons was trained. The training algorithm was gradient descent error back-propagation (EBP) with momentum (α) and adaptive learning rate (η). In order to generalize the network, the early stopping strategy and a 10-fold permuted cross-validation technique was applied which in each fold the data was divided into 80% training, 10% validation, and 10% testing sets. The network parameters were updated in the ‘incremental’ mode (each time an input is presented to the network), with random initial parameters in the range of [−0.5,0.5]. The network and the model were evaluated using Mean Square Error (MSE) and the coefficient of determination criteria of the training and test data. Coefficient of determination (or R-squared) is a statistical metric used in regression models that determines their goodness of fit. R-squared values close to 1 indicate that the model fits the data well. MSE is the most widely used metric to assess a network’s performance. A properly-trained network exhibits not only small training error, but also its test error falls within the range of training error. The evaluation measures for present study can be found in Supplemental Tab. 37.

To extract effective connectivity from the network’s input to its output, the Taylor expansion of the hidden neurons’ activation function was used to segregate the linear and nonlinear parts of $f\left(.\right)$:

2 $$f={f}^{{Lin}}+{f}^{{NonLin}}$$

The Taylor expansion is a mathematical method that accurately approximates a function by summation of linear and nonlinear terms. Its linear terms are the first-degree polynomial terms and nonlinear terms are higher-degree polynomial terms. In the nCREANN method, ${f}^{{Lin}}$ is a part of the input-output mapping that only represent the linear interactions of the signals, while the nonlinear part, ${f}^{{NonLin}}$, contains the information about the nonlinear interactions of them.

Linear effective connectivity: Based on the ${f}^{{Lin}}$, Linear Connectivity $({{lC}}_{i\to j})$ is calculated by multiplication of the network’s connecting weights and the hidden neuron’s scaling parameters. It represents the linear influence of *i*th region on the *j*th region (for detailed information).

Nonlinear effective connectivity: Nonlinear Connectivity$\,({NC})$ of ${x}_{i}$ to ${x}_{j}\,$ is defined by the ratio of the network’s estimation errors (difference between original values and predicted values).

3 $${{NC}}_{i\to j}={ln}\left(\frac{\left\langle {{\left({\epsilon }_{j}\right)}_{{x}_{i}{{\_}}{Lin}}}^{2}\right\rangle }{\left\langle {\left({\epsilon }_{j}\right)}^{2}\right\rangle }\right)$$

At the numerator, ${x}_{i}$ has only linear effect on ${x}_{j}$, but other remaining input signals have both linear and nonlinear influence on ${x}_{j}$. The denominator is the estimation error when all the input signals have both linear and nonlinear influence on ${x}_{j}$. The ${{NC}}_{i\to j}$ determines how much ${x}_{i}$ has nonlinear causal effect on ${x}_{j}$.

When estimating effective connectivity through EEG signals, it is crucial to take into account the volume conduction issue (transmission of electrical activity from the brain through the conductive tissues (e.g., scalp, skull) and fluids (e.g., cerebrospinal fluid) to the electrodes placed on the scalp). Volume conduction may cause confounding connectivity results^100^. A common option is to evaluate the connectivity for the underlying sources reconstructed with an inverse method on EEG data. In the present study, the nCREANN method was applied on clusters defined by DBSCAN, and for each of these clusters, the time series data were reconstructed by LCMV method in each frequency band. A common spatial filter within each group was used for the LCMV running. For the network to be well-trained, we used all data points of each trial in the time interval from −500 to 500 ms and concatenated all trials to achieve a sufficient length of data. The optimum model order was evaluated using Akaike and Schwartz criteria^101^. These criteria suggested the model order 7 to 10 for different frequency bands in the adult and adolescent groups. In the present study, the optimum model order $p=10$ was considered for both groups. As it was previously shown by Talebi et al.^55^, the assessment based on simulated data demonstrated that for time-lags (model order) greater than the actual values, the linear model’s coefficients are very close to 0, and the network efficiently detects that the signal samples at those time-lags are of no informative value. The same model order consideration has also been done in other studies as well^102–104^. This is shown in Supplemental Fig. 4.

The significance of the connectivity values was assessed through generation of 100 surrogate data with time-shifted surrogate method^105,106^. This method destroys any causal effect of between the signals but keeps the internal dynamics of each time series. The network settings were exactly the same as those used for the original data when using nCREANN to the surrogate data. The connectivity patterns are plotted on a typical head for significant connections that exceeded the 90% of the surrogate data. The coordinates of the source clusters are the same as in the DICS analysis (Beamforming Analyses section). Connectivity arrows show the information flow from one cluster to another one, while the arrow thickness and the size of the arrowhead is proportional to the connectivity strength. For the linear connectivity patterns, the mean absolute values of all non-self linear measures are averaged for all time lag and all subjects in each frequency band and each group. The nonlinear connectivity plots are based on the non-self nonlinear measures averaged over all subjects within each group in each frequency band. The nCREANN method was applied as recently published^104^.

### Correlational analysis

In order to assess the relationship between behavioral performance and neurophysiological data, we utilized partial correlation analysis using the MATLAB function ‘partialcorr’. The behavioral data included logistic regression coefficients from the first logistic regression model assigned to each participant, reflecting their sensitivity to changes in the situation count. On the other hand, the neurophysiological data encompassed the connectivity findings between the clusters as derived from nCREANN, which indicated the interaction between distinct brain regions. Following the partial correlation analyses, we employed False Discovery Rate (FDR) correction using the MATLAB function ‘mafdr’ to account for multiple comparisons.

### Statistics and reproducibility

The sample size is comparable to previous work using the same task and electrophysiological (EEG) methods^38^. EEG data analyses used cluster-based permutation testing to control for type 1 error (i.e. corrections for multiple comparisons). The analysis of network connectivity patterns is presented also using surrogate data (see above). Correlational analyses results were FDR corrected (see above). We provide measures of effect sizes (i.e. ORs) in the statistical analysis. Source localization and network connectivity analyses were also conducted for a comparison condition to ensure the specificity of the obtained results (Supplemental Figs. 1, 2 and 3). All data is available in OSF.

### Supplementary information

Supplemental Information

## Data Availability

Raw data can be found in OSF (10.17605/OSF.IO/M7V2P)^107^ The numerical source data for Fig. 1(a) and (b) and be found in the scripts that have been provided in OSF under the name 03_code behavioral analysis, A4 script.

## Code availability

Custom code used to process the data can be found in OSF (10.17605/OSF.IO/M7V2P)^107^.
