# Supplementary material for: Neural connectivity patterns explain why adolescents perceive the world as moving slow
Source: Commun Biol. 2024 Jun 22;7:759. doi: 10.1038/s42003-024-06439-4 (PMC11193795; doi:10.1038/s42003-024-06439-4)
Supplement: Supplementary file 1 — Supplemental Information [file 42003_2024_6439_MOESM1_ESM.pdf]

Supplementary Information

Neural connectivity patterns explain why adolescents perceive the world as moving slow

F. Ghorbani, X. Zhou, N. Talebi, V. Roessner, B. Hommel, A. Prochnow, C. Beste

Results of nCREANN Analysis After Surrogate Testing: Linear Connectivity

All connectivity values were multiplied by 100 for a more comprehensible presentation. The significant connections (those that exceeded 90% of the surrogate data) were averaged across subjects in each group for linear connectivity.

Theta Frequency Band

Supplemental Table 1. *Linear Connectivity in Theta Frequency Band in Adults.*

To \ From	Left-IT	Left-FT	Right-TO	Left-TO	MF
Left-IT		4.130	2.211	2.337	2.356
Left-FT	3.513		1.721	2.502	1.993
Right-TO	1.604	1.811		2.396	1.748
Left-TO	2.026	2.813	2.310		1.748
MF	1.377	1.769	1.601	1.465	

Note. IT = inferior temporal cortex, FT = fronto-temporal cortex, TO = temporo-occipital cortex, MF = medial frontal cortex.

Supplemental Table 2. *Linear Connectivity in Theta Frequency Band in Adolescents.*

To \ From	Left-TO	Right-TO	Right-FT	MF
Left-TO		2.535	2.282	1.894
Right-TO	2.846		2.763	1.988
Right-FT	2.294	2.455		2.015
MF	1.632	1.659	1.900	

Note. TO = temporo-occipital cortex, FT = fronto-temporal cortex, MF = medial frontal cortex.

Alpha Frequency Band

Supplemental Table 3. *Linear Connectivity in Alpha Frequency Band in Adults.*

To \ From	Left-IT	Right-TO	Left-TO	MF
Left-IT		3.464	3.420	3.246
Right-TO	1.767		4.694	1.948
Left-TO	2.352	4.797		1.866
MF	1.942	2.178	1.912	

Note. IT = inferior temporal cortex, TO = temporo-occipital cortex, MF = medial frontal cortex.

Supplemental Table 4. *Linear Connectivity in Alpha Frequency Band in Adolescents.*

To \ From	Right-TO	Left-TO	MF
Right-TO		2.684	2.215
Left-TO	3.834		2.326
MF	2.994	2.208	

Note. TO = temporo-occipital cortex, MF = medial frontal cortex.

Beta Frequency Band

Supplemental Table 5. *Linear Connectivity in Beta Frequency Band in Adults.*

To \ From	Left-IT	Left-TO	MF
Left-IT		6.029	3.310
Left-TO	5.699		3.335
MF	3.633	3.814	

Note. IT = inferior temporal cortex, TO = temporo-occipital cortex.

Supplemental Table 6. *Linear Connectivity in Beta Frequency Band in Adolescents.*

To \ From	Left-IT	Right-TO	Right-FT	MF
Left-IT		4.043	3.666	3.780
Right-TO	4.194		10.812	3.905
Right-FT	3.735	11.517		4.436
MF	2.471	3.157	3.936	

Note. IT = inferior temporal cortex, TO = temporo-occipital cortex, FT = fronto-temporal cortex, MF = medial frontal cortex.

Results of nCREANN Analysis After Surrogate Testing: Non-Linear Connectivity

All connectivity values were multiplied by 100 for a more comprehensible presentation. The significant connections (those that exceeded 90% of the surrogate data) were averaged across subjects in each group for non-linear connectivity.

Theta Frequency Band

Supplemental Table 7. *Non-Linear Connectivity in Theta Frequency Band in Adults.*

To \ From	Left-IT	Left-FT	Right-TO	Left-TO	MF
Left-IT		4.932	2.230	3.521	1.579
Left-FT	4.987		2.802	3.570	2.409
Right-TO	0.628	0.488		1.059	0.131
Left-TO	1.691	1.920	1.312		0.547
MF	-0.031	-0.083	0.033	-0.319	

Note. IT = inferior temporal cortex, FT = fronto-temporal cortex, TO = temporo-occipital cortex, MF = medial frontal cortex.

Supplemental Table 8. *Non-Linear Connectivity in Theta Frequency Band in Adolescents.*

To \ From	Left-TO	Right-TO	Right-FT	MF
Left-TO		1.424	1.636	0.480
Right-TO	1.557		1.013	0.361
Right-FT	0.249	0.234		-0.169
MF	-0.030	-0.299	-0.228	

Note. TO = temporo-occipital cortex, FT = fronto-temporal cortex, MF = medial frontal cortex.

Alpha Frequency Band

Supplemental Table 9. *Non-Linear Connectivity in Alpha Frequency Band in Adults.*

To \ From	Left-IT	Right-TO	Left-TO	MF
Left-IT		2.981	3.902	3.623
Right-TO	1.871		2.993	2.021
Left-TO	1.870	3.923		1.985
MF	1.105	0.836	0.763	

Note. IT = inferior temporal cortex, TO = temporo-occipital cortex, MF = medial frontal cortex.

Supplemental Table 10. *Non-Linear Connectivity in Alpha Frequency Band in Adolescents.*

To \ From	Right-TO	Left-TO	MF
Right-TO		2.747	2.847
Left-TO	2.260		2.105
MF	2.685	1.562	

Note. TO = temporo-occipital cortex, MF = medial frontal cortex.

Beta Frequency Band

Supplemental Table 11. *Non-Linear Connectivity in Beta Frequency Band in Adults.*

To \ From	Left-IT	Left-TO	MF
Left-IT		5.784	2.403
Left-TO	4.401		2.647
MF	2.298	2.574	

Note. IT = inferior temporal cortex, TO = temporo-occipital cortex.

Supplemental Table 12. *Non-Linear Connectivity in Beta Frequency Band in Adolescents.*

To \ From	Left-TO	Right-TO	Right-FT	MF
Left-TO		2.020	1.376	0.607
Right-TO	4.255		10.450	4.310
Right-FT	3.835	9.724		5.449
MF	0.574	1.177	1.322	

Note. TO = temporo-occipital cortex, FT = fronto-temporal cortex, MF = medial frontal cortex.

Results of nCREANN Analysis Without Surrogate Testing: Linear Connectivity

All connectivity values were multiplied by 100 for a more comprehensible presentation.

Theta Frequency Band

Supplemental Table 13. *Linear Connectivity in Theta Frequency Band in Adults.*

To \ From	Left-IT	Left-FT	Right-TO	Left-TO	MF
Left-IT		4.085	2.202	2.351	2.314
Left-FT	3.457		1.691	2.421	1.964
Right-TO	1.588	1.784		2.372	1.727
Left-TO	2.003	2.747	2.300		1.764
MF	1.348	1.762	1.531	1.456	

Note. IT = inferior temporal cortex, FT = fronto-temporal cortex, TO = temporo-occipital cortex, MF = medial frontal cortex.

Supplemental Table 14. Linear Connectivity in Theta Frequency Band in Adolescents.

To \ From	Left-TO	Right-TO	Right-FT	MF
Left-TO		2.644	2.222	1.912
Right-TO	2.753		2.867	1.963
Right-FT	2.354	2.537		2.108
MF	1.660	1.722	1.795	

Note. TO = temporo-occipital cortex, FT = fronto-temporal cortex, MF = medial frontal cortex.

Alpha Frequency Band

Supplemental Table 15. *Linear Connectivity in Alpha Frequency Band in Adults.*

To \ From	Left-IT	Right-TO	Left-TO	MF
Left-IT		3.295	3.315	3.066
Right-TO	1.726		4.694	1.823
Left-TO	2.266	4.797		1.856
MF	1.877	2.019	1.820	

Note. IT = inferior temporal cortex, TO = temporo-occipital cortex, MF = medial frontal cortex.

Supplemental Table 16. *Linear Connectivity in Alpha Frequency Band in Adolescents.*

To \ From	Right-TO	Left-TO	MF
Right-TO		2.796	2.243
Left-TO	3.610		2.307
MF	2.905	2.214	

Note. TO = temporo-occipital cortex, MF = medial frontal cortex.

Beta Frequency Band

Supplemental Table 17. *Linear Connectivity in Beta Frequency Band in Adults.*

To \ From	Left-IT	Left-TO	MF
Left-IT		5.735	3.179
Left-TO	5.699		3.218
MF	3.633	3.554	

Note. IT = inferior temporal cortex, TO = temporo-occipital cortex.

Supplemental Table 18. *Linear Connectivity in Beta Frequency Band in Adolescents.*

To \ From	Left-IT	Right-TO	Right-FT	MF
Left-IT		3.964	3.530	3.584
Right-TO	4.081		10.812	3.795
Right-FT	3.492	11.325		4.349
MF	2.360	3.122	3.710	

Note. IT = inferior temporal cortex, TO = temporo-occipital cortex, FT = fronto-temporal cortex, MF = medial frontal cortex.

Results of nCREANN Analysis Without Surrogate Testing: Non-Linear Connectivity

All connectivity values were multiplied by 100 for a more comprehensible presentation. The average of all non-linear connectivity values used as threshold was at 1.569.

Theta Frequency Band

Supplemental Table 19. *Non-Linear Connectivity in Theta Frequency Band in Adults.*

To \ From	Left-IT	Left-FT	Right-TO	Left-TO	MF
Left-IT		4.555	1.846	2.888	1.222
Left-FT	5.122		2.972	3.404	2.316
Right-TO	0.528	0.399		0.740	0.095
Left-TO	1.438	1.433	1.180		0.429
MF	-0.043	-0.032	0.015	-0.293	

Note. IT = inferior temporal cortex, FT = fronto-temporal cortex, TO = temporo-occipital cortex, MF = medial frontal cortex.

Supplemental Table 20. Non-Linear Connectivity in Theta Frequency Band in Adolescents.

To \ From	Left-TO	Right-TO	Right-FT	MF
Left-TO		1.214	1.295	0.396
Right-TO	1.480		1.191	0.361
Right-FT	0.243	0.100		-0.208
MF	-0.038	-0.266	-0.207	

Note. TO = temporo-occipital cortex, FT = fronto-temporal cortex, MF = medial frontal cortex.

Alpha Frequency Band

Supplemental Table 21. *Non-Linear Connectivity in Alpha Frequency Band in Adults.*

To \ From	Left-IT	Right-TO	Left-TO	MF
Left-IT		2.700	3.236	2.929
Right-TO	1.794		2.993	1.714
Left-TO	1.782	3.422		1.602
MF	0.876	0.626	0.547	

Note. IT = inferior temporal cortex, TO = temporo-occipital cortex, MF = medial frontal cortex.

Supplemental Table 22. *Non-Linear Connectivity in Alpha Frequency Band in Adolescents.*

To \ From	Right-TO	Left-TO	MF
Right-TO		2.210	2.570
Left-TO	1.869		1.949
MF	2.170	1.759	

Note. TO = temporo-occipital cortex, MF = medial frontal cortex.

Beta Frequency Band

Supplemental Table 23. *Non-Linear Connectivity in Beta Frequency Band in Adults.*

To \ From	Left-IT	Left-TO	MF
Left-IT		5.514	2.360
Left-TO	4.183		2.446
MF	2.325	2.387	

Note. IT = inferior temporal cortex, TO = temporo-occipital cortex.

Supplemental Table 24. *Non-Linear Connectivity in Beta Frequency Band in Adolescents.*

To \ From	Left-TO	Right-TO	Right-FT	MF
Left-TO		2.020	1.376	0.607
Right-TO	4.255		10.450	4.310
Right-FT	3.835	9.724		5.449
MF	0.574	1.177	1.322	

Note. TO = temporo-occipital cortex, FT = fronto-temporal cortex, MF = medial frontal cortex.

Pattern of Connectivity Between Different Brain Regions Without Surrogate Testing

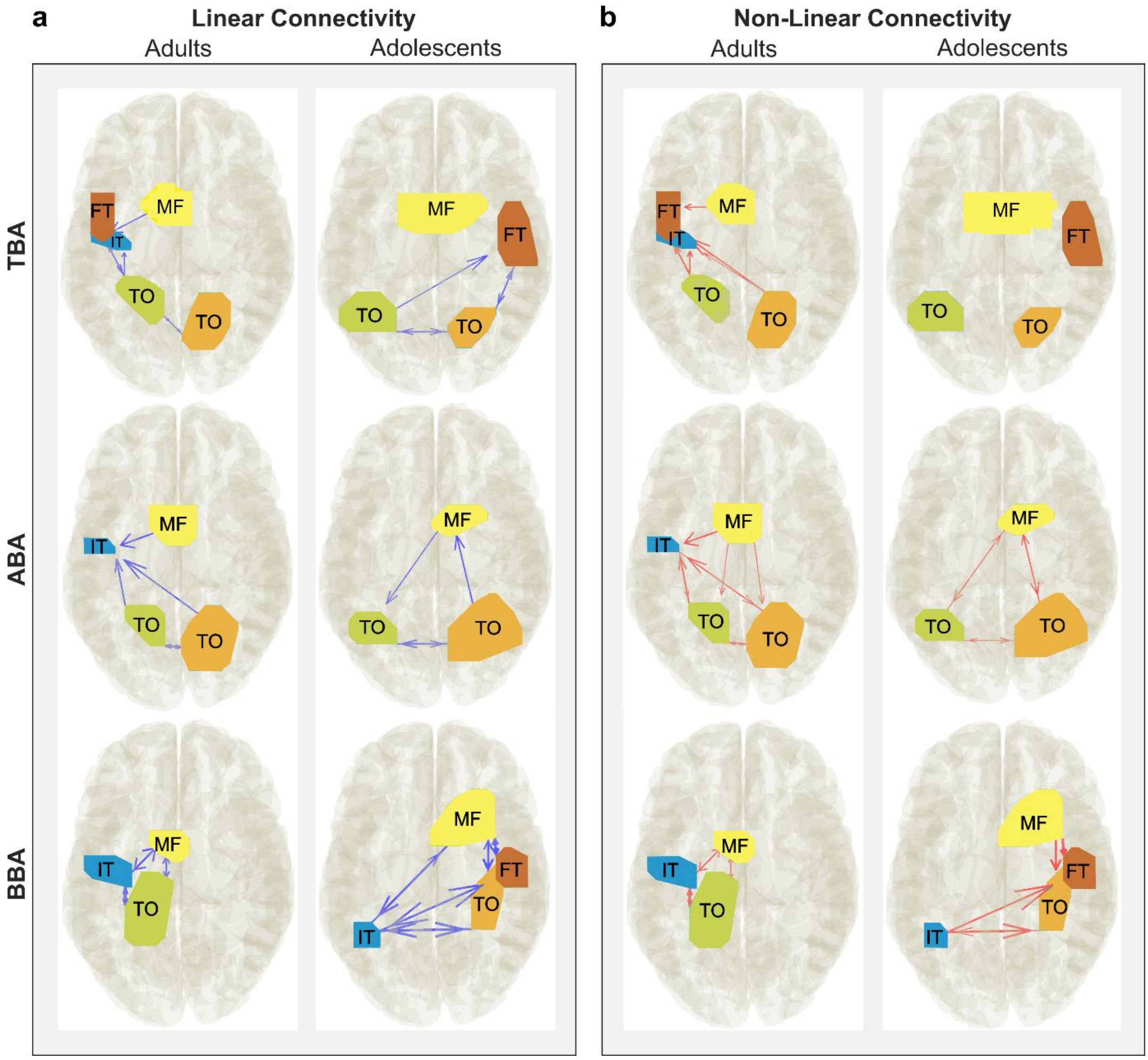

Supplemental Fig. 1 – nCREANN Connectivity for Adults and Adolescents. The nCREANN Linear and Non-Linear connectivity between brain regions for each frequency band before the surrogate testing. The blue arrows in figure part (a) represent Linear connectivity and the red arrows in figure part (b) represent Non-Linear connectivity. IT = inferior temporal cortex, FT = fronto-temporal cortex, TO = temporo-occipital cortex, MF = medial frontal cortex.

Results of Partial Correlational Analysis Between Connectivity Values and Behavioral

Theta Frequency Band

Supplemental Table 25. *Partial Correlations of the Behavioral Segmentation Probability with the Linear Connectivity in Theta Frequency Band in Adults.*

To \ From	Left-IT		Left-FT		Right-TO		Left-TO		MF	
	r	p	r	p	r	p	r	p	r	p
Left-IT			-0.064	0.781	-0.167	0.470	-0.044	0.850	0.006	0.980
Left-FT	0.459	0.036			0.094	0.686	-0.162	0.482	-0.087	0.709
Right-TO	-0.055	0.814	-0.343	0.128			0.052	0.823	-0.105	0.650
Left-TO	-0.152	0.511	0.064	0.782	0.056	0.810			0.215	0.349
MF	-0.117	0.613	-0.049	0.833	0.111	0.633	0.101	0.664		

Note. IT = inferior temporal cortex, FT = fronto-temporal cortex, TO = temporo-occipital cortex, MF = medial frontal cortex.

Supplemental Table 26. *Partial Correlations of the Behavioral Segmentation Probability with the Linear Connectivity in Theta Frequency Band in Adolescents.*

To \ From	Left-TO		Right-TO		Right-FT		MF	
	r	p	r	p	r	p	r	p
Left-TO			0.347	0.071	-0.280	0.149	-0.142	0.470
Right-TO	-0.168	0.393			0.237	0.224	-0.060	0.761
Right-FT	0.201	0.305	0.110	0.576			0.068	0.732
MF	-0.091	0.646	0.174	0.377	0.139	0.480		

Note. TO = temporo-occipital cortex, FT = fronto-temporal cortex, MF = medial frontal cortex.

Supplemental Table 27. *Partial Correlations of the Behavioral Segmentation Probability with the Non-Linear Connectivity in Theta Frequency Band in Adults.*

To \ From	Left-IT		Left-FT		Right-TO		Left-TO		MF	
	r	p	r	p	r	p	r	p	r	p
Left-IT			-0.401	0.071	0.451	0.040	0.005	0.981	0.150	0.517
Left-FT	-0.059	0.801			0.232	0.312	-0.124	0.593	0.145	0.530
Right-TO	-0.449	0.041	-0.205	0.372			0.256	0.263	0.444	0.044
Left-TO	0.060	0.796	-0.230	0.316	0.309	0.172			-0.137	0.553
MF	0.417	0.060	0.247	0.281	-0.597	0.004	-0.113	0.627		

Note. IT = inferior temporal cortex, FT = fronto-temporal cortex, TO = temporo-occipital cortex, MF = medial frontal cortex.

Supplemental Table 28. *Partial Correlations of the Behavioral Segmentation Probability with the Non-Linear Connectivity in Theta Frequency Band in Adolescents.*

To \ From	Left-TO		Right-TO		Right-FT		MF	
	r	p	r	p	r	p	r	p
Left-TO			-0.137	0.486	0.117	0.552	0.087	0.661
Right-TO	0.251	0.197			-0.270	0.164	-0.136	0.492
Right-FT	-0.397	0.036	0.236	0.227			0.404	0.033
MF	0.015	0.942	0.075	0.705	0.001	0.997		

Note. TO = temporo-occipital cortex, FT = fronto-temporal cortex, MF = medial frontal cortex.

Alpha Frequency Band

Supplemental Table 29. *Partial Correlations of the Behavioral Segmentation Probability with the Linear Connectivity in Alpha Frequency Band in Adults.*

To \ From	Left-IT		Right-TO		Left-TO		MF	
	r	p	r	p	r	p	r	p
Left-IT			-0.110	0.571	0.222	0.248	-0.230	0.231
Right-TO	0.372	0.047			0.102	0.598	-0.196	0.308
Left-TO	-0.196	0.308	-0.161	0.403			0.480	0.008
MF	0.372	0.047	0.054	0.782	-0.500	0.006		

Note. IT = inferior temporal cortex, TO = temporo-occipital cortex, MF = medial frontal cortex.

Supplemental Table 30. *Partial Correlations of the Behavioral Segmentation Probability with the Linear Connectivity in Alpha Frequency Band in Adolescents.*

To \ From	Right-TO		Left-TO		MF	
	r	p	r	p	r	p
Right-TO			0.197	0.265	0.166	0.348
Left-TO	-0.109	0.538			-0.033	0.854
MF	0.058	0.744	-0.199	0.260		

Note. TO = temporo-occipital cortex, MF = medial frontal cortex.

Supplemental Table 31. *Partial Correlations of the Behavioral Segmentation Probability with the Non-Linear Connectivity in Alpha Frequency Band in Adults.*

From \ To	Left-IT		Right-TO		Left-TO		MF	
	r	p	r	p	r	p	r	p
Left-IT			-0.022	0.911	-0.027	0.889	0.021	0.912
Right-TO	0.079	0.686			0.315	0.097	-0.168	0.384
Left-TO	0.071	0.713	-0.320	0.091			0.019	0.921
MF	-0.025	0.899	-0.185	0.336	0.367	0.050		

Note. IT = inferior temporal cortex, TO = temporo-occipital cortex, MF = medial frontal cortex.

Supplemental Table 32. *Partial Correlations of the Behavioral Segmentation Probability with the Non-Linear Connectivity in Alpha Frequency Band in Adolescents.*

To \ From	Right-TO		Left-TO		MF	
	r	p	r	p	r	p
Right-TO			0.003	0.988	0.042	0.813
Left-TO	-0.034	0.849			0.017	0.926
MF	0.127	0.474	-0.213	0.226		

Note. TO = temporo-occipital cortex, MF = medial frontal cortex.

Beta Frequency Band

Supplemental Table 33. *Partial Correlations of the Behavioral Segmentation Probability with the Linear Connectivity in Beta Frequency Band in Adults.*

From \ To	Left-IT		Left-TO		Left-TO	
	r	p	r	p	r	p
Left-IT			0.009	0.959	-0.095	0.588
Left-TO	-0.028	0.875			0.104	0.553
MF	0.066	0.708	0.114	0.514		

Note. IT = inferior temporal cortex, TO = temporo-occipital cortex.

Supplemental Table 34. *Partial Correlations of the Behavioral Segmentation Probability with the Linear Connectivity in Beta Frequency Band in Adolescents.*

To \ From	Left-IT		Right-TO		Right-FT		MF	
	r	p	r	p	r	p	r	p
Left-IT			-0.142	0.472	0.087	0.659	-0.088	0.658
Right-TO	0.362	0.058			-0.309	0.109	0.159	0.419
Right-FT	-0.152	0.441	0.216	0.270			-0.013	0.948
MF	0.098	0.620	-0.244	0.212	0.269	0.166		

Note. IT = inferior temporal cortex, TO = temporo-occipital cortex, FT = fronto-temporal cortex, MF = medial frontal cortex.

Supplemental Table 35. *Partial Correlations of the Behavioral Segmentation Probability with the Non-Linear Connectivity in Beta Frequency Band in Adults.*

To \ From	Left-IT		Left-TO		Left-TO	
	r	p	r	p	r	p
Left-IT			0.049	0.780	0.062	0.721
Left-TO	-0.106	0.544			0.142	0.416
MF	-0.096	0.582	0.133	0.445		

Note. IT = inferior temporal cortex, TO = temporo-occipital cortex.

Supplemental Table 36. *Partial Correlations of the Behavioral Segmentation Probability with the Non-Linear Connectivity in Beta Frequency Band in Adolescents.*

From / To	Left-TO		Right-TO		Right-FT		MF	
	r	p	r	p	r	p	r	p
Left-TO			-0.079	0.690	0.115	0.559	-0.007	0.974
Right-TO	0.287	0.139			0.034	0.862	-0.102	0.606
Right-FT	-0.301	0.119	0.017	0.930			0.050	0.801
MF	-0.205	0.294	0.094	0.634	0.241	0.216		

Note. TO = temporo-occipital cortex, FT = fronto-temporal cortex, MF = medial frontal cortex.

The Model Evaluation Measures of The Networks

Supplemental Table 37. *Evaluation Measures of The Network Model*

		Theta	Alpha	Beta
Adolescents	R_squared	.896 ± .021	.881 ± .062	.883 ± .024
	MSE train	.073 ± .024	.070 ± .020	.064 ± .012
	MSE test	.032 ± .010	.030 ± .016	.042 ± .009
Adults	R_squared	.902 ± .009	.878 ± .110	.890 ± .010
	MSE train	.071 ± .024	.059 ± .019	.050 ± .010
	MSE test	.031 ± .011	.021 ± .014	.028 ± .008

Note. The coefficient of determination the R-Squared and the Mean Square Error (MSE) of the training and test data. The values are Mean ± Standard Deviation across all subjects.

Source Localization Comparison Between Boundary and No-boundary Time Intervals

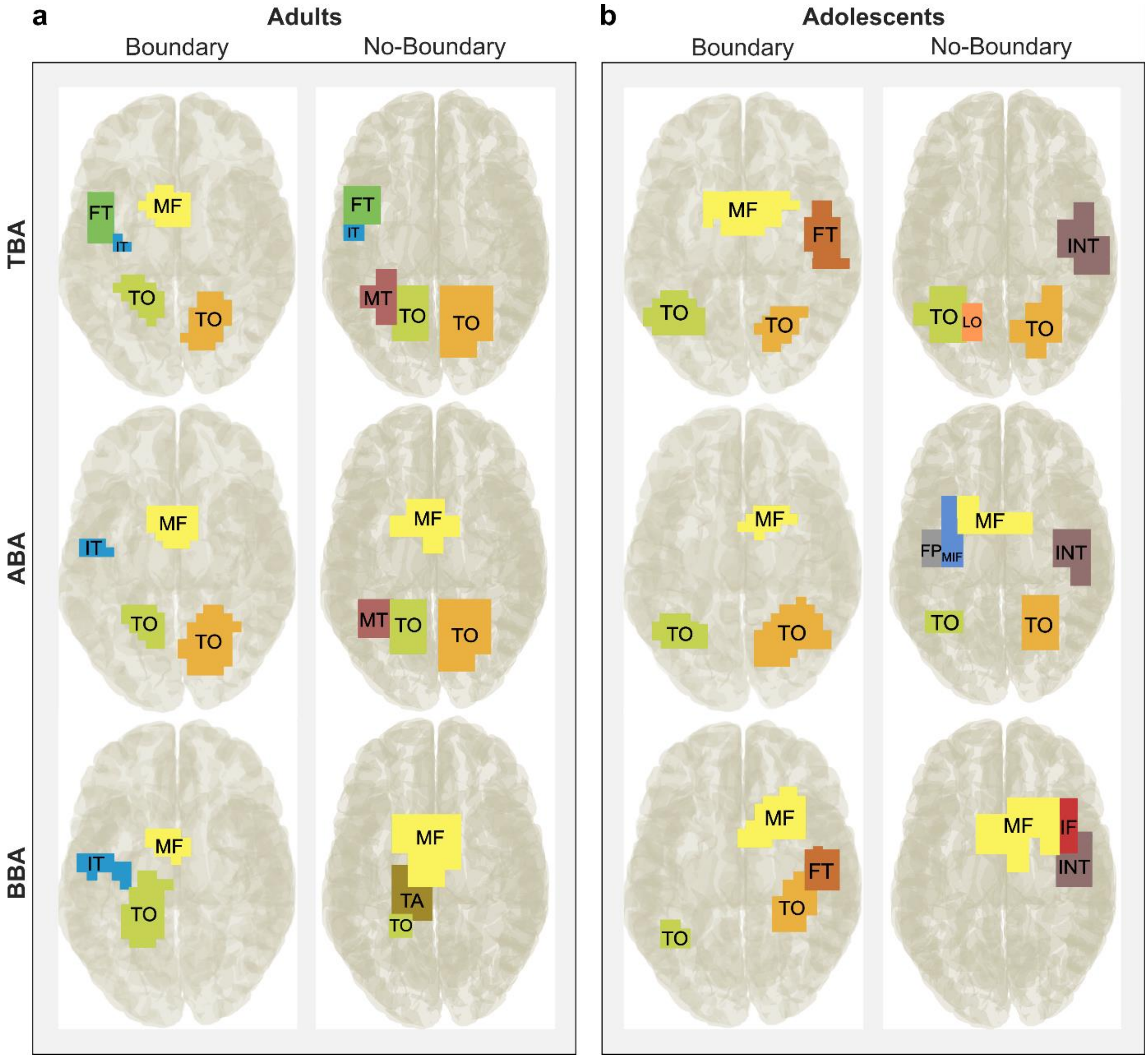

Supplemental Fig. 2 – DBSCAN Results for Adults and Adolescents. Results of the DBSCAN source localization algorithm for comparing Boundary interval with the No-Boundary interval in both groups of (a) adults and (b) adolescents. IT = inferior temporal cortex, FT = fronto-temporal cortex, TO = temporo-occipital cortex, MF = medial frontal cortex, MT = medial temporal cortex, TA= thalamus, LO = lateral occipital cortex, INT = insulo-temporal cortex, FP = fronto-parietal cortex, MIF = middle frontal cortex, IF = inferior frontal cortex. Please note that thalamic structures should be considered with caution due to the low in-depth resolution of EEG-beamforming

Results of nCREANN Analysis After Surrogate Testing for No Boundary Interval (NBI): Linear Connectivity

All connectivity values were multiplied by 100 for a more comprehensible presentation. The significant connections (those that exceeded 90% of the surrogate data) were averaged across subjects in each group for linear connectivity.

Theta Frequency Band

Supplemental Table 38. *Linear Connectivity in Theta Frequency Band in Adults.*

To \ From	Left-IT	Right-TO	Left-TO	Left-MT	Left-FT
Left-IT		2.829	2.634	2.500	2.210
Right-TO	1.904		3.626	2.539	1.748
Left-TO	2.234	3.752		3.747	2.307
Left-MT	2.424	2.732	3.849		2.635
Left-FT	2.398	2.490	2.959	2.500	

Note. IT = inferior temporal cortex, TO = temporo-occipital cortex, MT = medial temporal cortex, FT = fronto-temporal cortex.

Supplemental Table 39. *Linear Connectivity in Theta Frequency Band in Adolescents.*

To \ From	Left-TO	Right-TO	Left-LO	Left-INT
Left-TO		2.443	3.626	1.980
Right-TO	2.432		3.112	2.093
Left-LO	3.688	2.935		1.697
Left-INT	2.313	2.351	2.200	

Note. TO = temporo-occipital cortex, LO = lateral occipital cortex, INT = insulo-temporal cortex.

Alpha Frequency Band

Supplemental Table 40. *Linear Connectivity in Alpha Frequency Band in Adults.*

To \ From	Right-TO	Left-TO	Left-MT	MF
Right-TO		4.115	2.400	2.379
Left-TO	3.736		5.538	2.563
Left-MT	2.656	5.982		2.449
MF	1.477	1.907	1.618	

Note. TO = temporo-occipital cortex, MT = medial temporal cortex, MF = medial frontal cortex.

Supplemental Table 41. *Linear Connectivity in Alpha Frequency Band in Adolescents.*

To \ From	Left-TO	Right-TO	Right-INT	Left-FP	Left-MIF	MF
Left-TO		2.362	2.233	2.526	3.305	2.417
Right-TO	2.120		2.883	2.030	2.892	1.950
Right-INT	1.676	2.784		2.394	2.838	2.016
Left-FP	1.737	1.704	2.158		5.631	2.810
Left-MIF	1.351	1.532	2.292	4.970		4.499
MF	1.688	1.570	2.077	2.490	5.572	

Note. TO = temporo-occipital cortex, INT = insulo-temporal cortex, FP = fronto-parietal cortex, MIF = middle frontal cortex, MF = medial frontal cortex.

Beta Frequency Band

Supplemental Table 42. *Linear Connectivity in Beta Frequency Band in Adults.*

To \ From	Left-TO	Left-TA	MF
Left-TO		10.386	2.968
Left-TA	10.361		5.069
MF	2.569	4.285	

Note. TO = temporo-occipital cortex, TA = thalamus, MF = medial frontal cortex.

Supplemental Table 43. *Linear Connectivity in Beta Frequency Band in Adolescents.*

To \ From	Right-INT	Right-IF	MF
Right-INT		4.043	3.785
Right-IF	3.030		4.692
MF	2.769	5.475	

Note. INT = insulo-temporal cortex, IF = inferior frontal cortex, MF = medial frontal cortex.

Results of nCREANN Analysis After Surrogate Testing for No-Boundary Interval (NBI): Non-Linear Connectivity

All connectivity values were multiplied by 100 for a more comprehensible presentation. The significant connections (those that exceeded 90% of the surrogate data) were averaged across subjects in each group for non-linear connectivity.

Theta Frequency Band

Supplemental Table 44. *Non-Linear Connectivity in Theta Frequency Band in Adults.*

To \ From	Left-IT	Right-TO	Left-TO	Left-MT	Left-FT
Left-IT		1.764	6.104	4.394	4.868
Right-TO	2.324		8.380	7.912	3.366
Left-TO	1.827	3.448		8.229	2.351
Left-MT	3.520	5.328	11.870		4.482
Left-FT	5.160	4.825	6.301	7.777	

Note. IT = inferior temporal cortex, TO = temporo-occipital cortex, MT = medial temporal cortex, FT = fronto-temporal cortex.

Supplemental Table 45. *Non-Linear Connectivity in Theta Frequency Band in Adolescents.*

To \ From	Left-TO	Right-TO	Left-LO	Right-INT
Left-TO		7.177	11.204	3.443
Right-TO	11.004		17.609	5.086
Left-LO	9.187	11.269		2.519
Right-INT	8.382	12.028	11.507	

Note. TO = temporo-occipital cortex, LO = lateral occipital cortex, INT = insulo-temporal cortex.

Alpha Frequency Band

Supplemental Table 46. *Non-Linear Connectivity in Alpha Frequency Band in Adults.*

To \ From	Right-TO	Left-TO	Left-MT	MF
Right-TO		10.146	2.777	-0.062
Left-TO	2.310		8.237	0.327
Left-MT	1.596	12.116		0.017
MF	0.210	4.359	2.076	

Note. TO = temporo-occipital cortex, MT= medial temporal cortex, MF = medial frontal cortex.

Supplemental Table 47. *Non-Linear Connectivity in Alpha Frequency Band in Adolescents.*

To \ From	Left-TO	Right-TO	Right-INT	Left-FP	Left-MIF	MF
Left-TO		3.014	1.702	0.802	1.733	1.395
Right-TO	0.996		2.123	1.278	1.719	1.180
Right-INT	1.111	2.495		0.730	1.898	0.759
Left-FP	0.152	1.837	0.754		2.793	1.892
Left-MIF	0.156	0.886	0.181	2.124		2.445
MF	0.562	1.427	0.967	0.858	3.673	

Note. TO = temporo-occipital cortex, INT = insulo-temporal cortex, FP = fronto-parietal cortex, MIF = middle frontal cortex, MF = medial frontal cortex.

Beta Frequency Band

Supplemental Table 48. *Non-Linear Connectivity in Beta Frequency Band in Adults.*

To \ From	Left-TO	Left-TA	MF
Left-TO		11.357	2.592
Left-TA	23.017		1.700
MF	10.160	7.556	

Note. TO = temporo-occipital cortex, TA = thalamus, MF = medial frontal cortex.

Supplemental Table 49. *Non-Linear Connectivity in Beta Frequency Band in Adolescents.*

To \ From	Right-INT	Right-IF	MF
Right-INT		7.176	3.837
Right-IF	8.346		4.227
MF	6.559	6.192	

Note. INT = insulo-temporal cortex, IF = inferior frontal cortex, MF = medial frontal cortex.

Pattern of Connectivity Between Different Brain Regions for No-Boundary Interval (NBI)

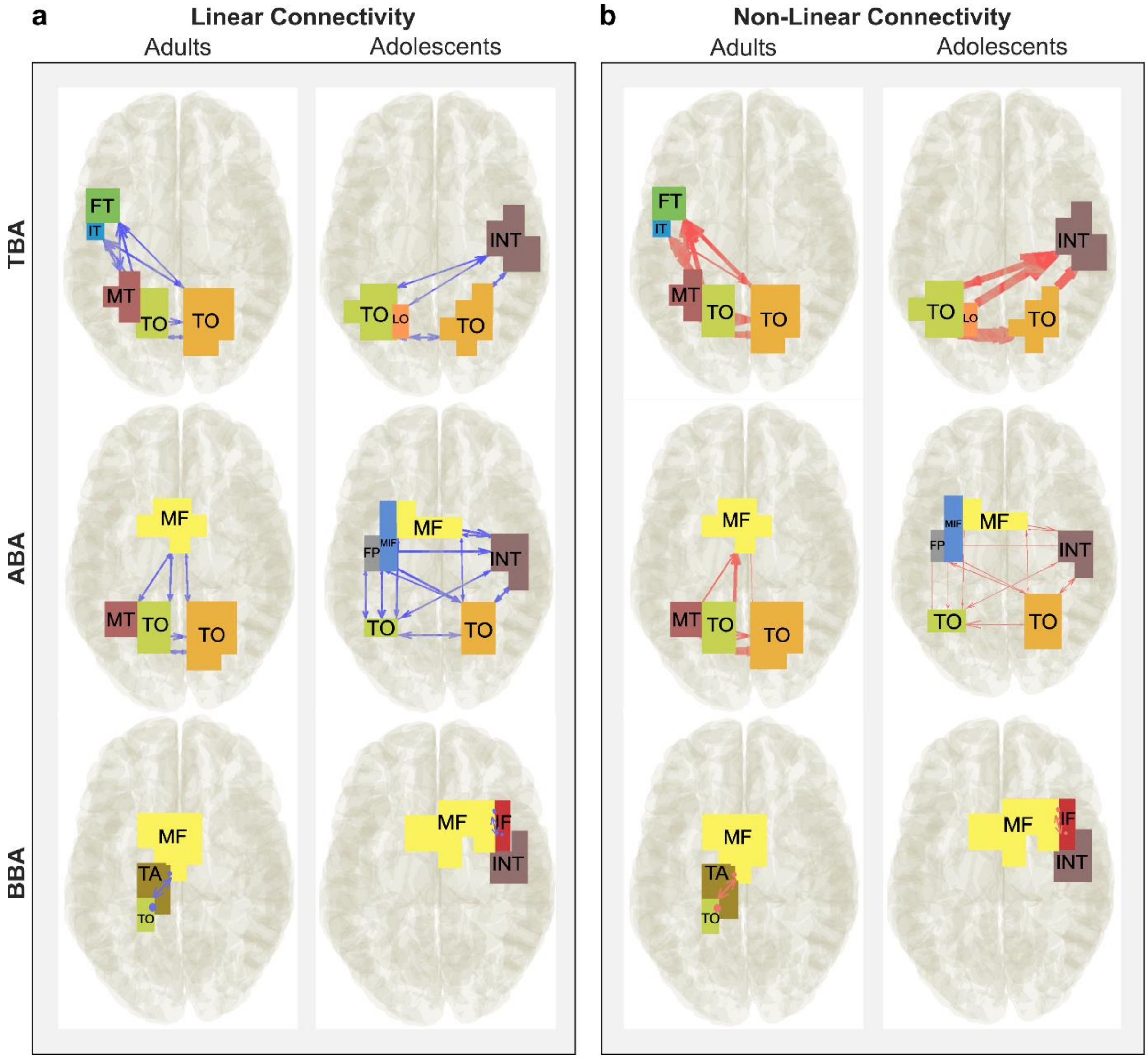

Supplemental Fig. 3 – nCREANN Connectivity for Adults and Adolescents for NBI. The nCREANN (a) Linear and (b) Non-Linear connectivity between brain regions for each frequency band for NBI. The blue arrows represent Linear connectivity and the red represents Non-Linear connectivity. IT = inferior temporal cortex, FT = fronto-temporal cortex, TO = temporo-occipital cortex, MF = medial frontal cortex, MT = medial temporal cortex, TA= thalamus, LO = lateral occipital cortex, INT = insulo-temporal cortex, FP = fronto-parietal cortex, MIF = middle frontal cortex, IF = inferior frontal cortex. Please note that thalamic structures should be considered with caution due to the low in-depth resolution of EEG-beamforming.

The model order for Adults and Adolescents for Boundary Interval (BI)

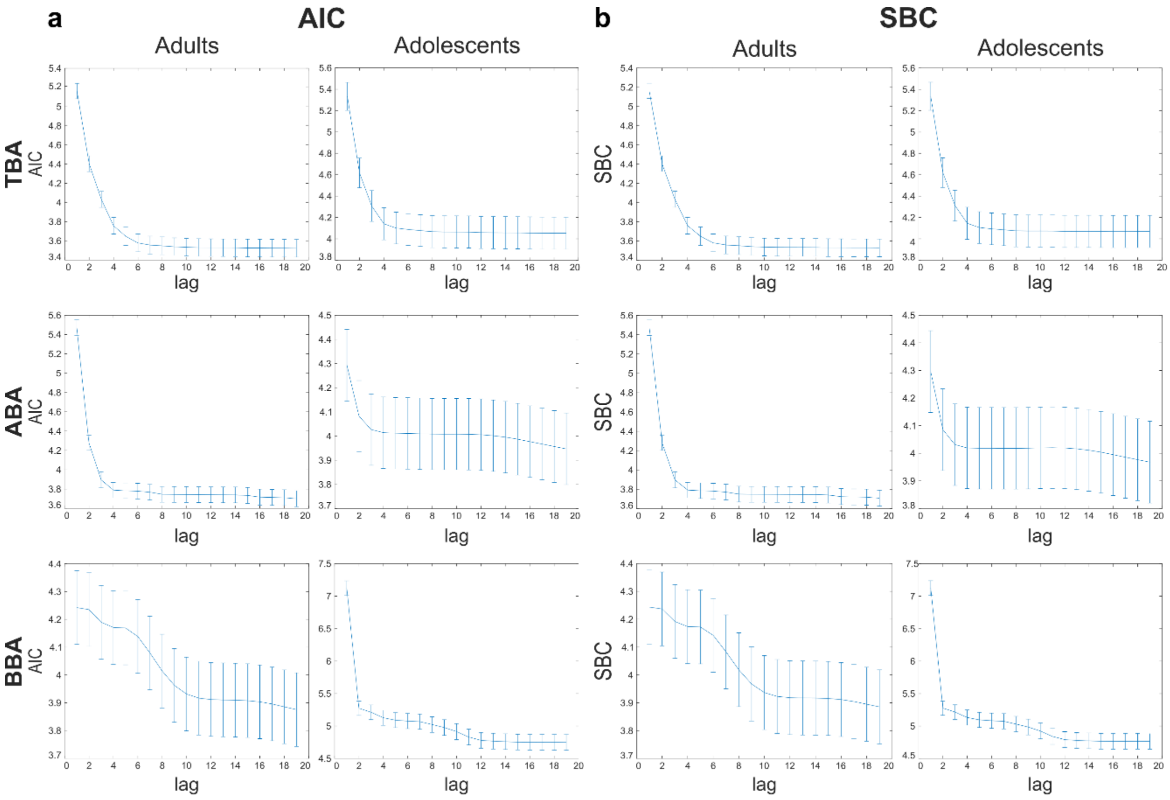

Supplemental Fig. 4 – The model order for Adults and Adolescents for BI. (a) AIC and (b) SBC criteria were computed for the model order estimation for theta, alpha, and beta frequency bands in adult and adolescent groups. The curves are Mean ± CI for all subjects (CI is 95% of confidence interval). The model order 10 was considered for all conditions.
